# Performance Review of Intelligent Guidance Robot at the Outpatient Clinic Setting

**DOI:** 10.7759/cureus.16840

**Published:** 2021-08-02

**Authors:** Alfred C Ma, Zuowei Meng, Xiaorong Ding

**Affiliations:** 1 Anesthesiology, Mansfield International College, Fullerton, USA; 2 Surgery, Peking University Shenzhen Hospital, Shenzhen, CHN; 3 Internal Medicine, Peking University Shenzhen Hospital, Shenzhen, CHN

**Keywords:** outpatient clinic, robot, guidance and counselling, information technology, health care literacy

## Abstract

This study analyzes the implementation of a mobile intelligent guidance robot to roam hospital outpatient services and discusses the application’s effect and experience. The data consist of human-robot verbal communications in November 2019 to analyze and evaluate the application according to the service volume, accuracy, and functions. Statistically, the accuracy of correct output by the intelligent guidance robot when answering related questions in outpatient services was significantly lower than the manufacturer’s claimed expected accuracy. Furthermore, the utilization review of the intelligent guidance robot was surprisingly unexpected. Therefore, applying an intelligent guidance robot is not limited to merely providing directions and navigation functions but can be valuable in improving public health literacy. Nevertheless, the hospital should meet patients’ needs by expanding intelligent guidance robots’ service functions and increasing patient experience to finetune the application through further experiments and design.

## Introduction

Continuous development of medical informatics prompted health care organizations to transform the traditional business practice to an artificial intelligence-savvy “smart hospital” model [1]. Patient intelligence service is an essential construct of an innovative hospital. It requires hospitals to use information technology such as medical service robots to provide more convenient, fast, and accurate medical services [2]. Outpatient medical service is an area with a high impact on the patient experience, and it is also portraying the image as organizational patient-centered management efficiencies. Implementing an intelligent guidance robot is one way for medical health care organizations to realize the transformation.

Health care practice varies across systems and cultures. In China, for example, a health care system that is heavily specialized, patients access health care services without a referral from a family doctor and do not know what specialty or provider they need to visit before arriving in the hospital’s lobby. Outpatient service continues to be an integral part of hospital practice in China. Navigating the complex outpatient schedules and specialties requires the help of nursing personnel to direct the patient to clinics and departments as they view appropriately [3]. Triaging patients upon arrival becomes a critical function at a hospital and indicates operational efficiencies and patient satisfaction.

Triaging of patients at health care organizations’ lobbies in China is unique. It is in high demand, high mobility, and highly repetitive. For this reason, health care organizations are introducing intelligent guidance robots (IGRs) in an attempt to ease the burden of traditional human-human interactions. Based on the analysis of human-computer interaction data, this essay aims to evaluate the effectiveness of human-robot verbal communication and the performance accuracy of a mobile IGR at a tertiary hospital with almost 10,000 daily outpatient visits in Shenzhen, China.

## Materials and methods

In brief, the speech interaction content between patients and IGR is a standardized keyword-based natural language processing and analysis summary. An intelligent referral service system puts the cloud language library into the robot to communicate with the patient’s natural voice to answer various hospital-related business functions and disease-related information. The IGR can also learn independently. Information is recorded and saved during encounters and deep neural network machine learning is used to achieve self-renewal of the knowledge base to understand users’ needs progressively better. As a result, the system continues to improve the accuracy and intelligence of the service, a genuinely patient-centered innovation. There are many IGR manufacturers. To maintain research objectivity, the authors decided not to disclose the manufacture and model of the robot.

The IGR functions were intended to improve the patient’s self-serving utility and reduce human-to-human interactions at the hospital [4]. The business function can be broadly classified as intelligent navigation, intelligent triage, and intelligent education. In terms of intelligent navigation, the IGR uses voice interaction to provide accurate service locations to patients and recommends the best route on a high-precision map display for patients to enhance the utilization experience. The IGR integrates outpatient hospital business hours and doctors’ schedules in each clinical service in intelligent triage. The intelligent consultation service system also provides the essential business operation that the hospital is currently available for patients. It connects with the hospital scheduling system in real-time to realize accurate information symmetry to direct patients to specialties clinics and functional departments such as radiology and operating room. Lastly, the intelligent education function provides dynamic publicity of hospital and health information through keyword search. When the visitor encounter was idle, the IGR is broadcasting helpful health information to improve public health literacy. Information regarding hospital business operation is integrated into the intelligent referral service system, including outpatient and emergency service, inpatient registration, discharge procedure, reimbursement settlement, and many more.

One year after the official implementation of the IGR at the outpatient clinic locations, in November 2019, for this study, an IGR was deployed for 20 days continuously at the main lobby of outpatient clinic during business hours. The study collected the quantitative values and content of human-computer voice interaction for analysis. The study further analyzed the successfully answered questions and applied commercially available software to comb through the data, to perform descriptive statistics and classification accuracy analysis. The manufacturer claimed classification accuracy outputs were compared manually to the observed count of accurate answers provided by the robot. The study also reviewed the most common questions that the visitors asked.

## Results

With the continuous improvement of the system application, the business function has assumed some of the information communication needs of outpatient daily medical services. However, through the data analysis, the study found that the robot in a noisy and challenging environment continues to accept error data, thus affecting the accuracy of the answer. In addition, the data analysis indicated visitors found the robot amusing and inputted non-medical related language and questions, such as "can you hold hands?" and "do you have a girlfriend?" These types of interactions can be abusive to the efficiency of the intended service.

**Figure 1:**
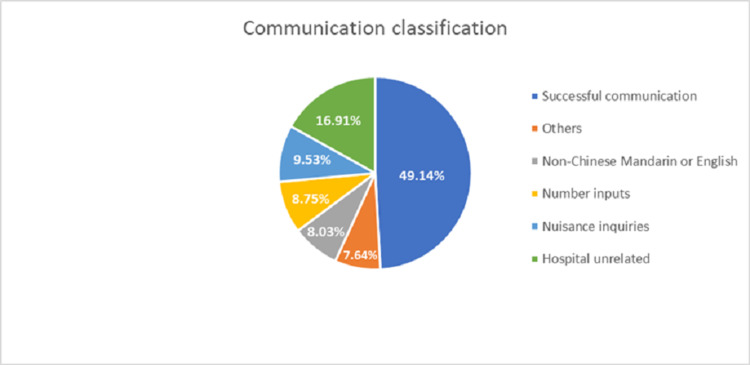
Communication classification during human-computer encounters

During the study period in November 2019, the outpatient IGR performed its duty from eight in the morning to eight in the evening daily. There were 150,851 outpatient visits recorded, and the study collected 25,526 human-robot encounters during the 20 days. In all, 16.92% of visitors utilized the IGR to seek information. There were 12,543 successful encounters in the study. A successful encounter is defined as the robot’s ability to provide an answer to a question. However, the robot failed to provide question-related answers in 13,183 encounters where the robot provided a default answer as "I am still learning. Please ask the nursing staff." Questions that the IGR could not answer were conveyed in languages other than Chinese Mandarin and English, numbers, child play or nuisance inquiries, and questions unrelated to hospital business and medical needs. Figure 1 depicts the percentage of communication success compared to those that the IGR could not process and answer.

**Figure 2:**
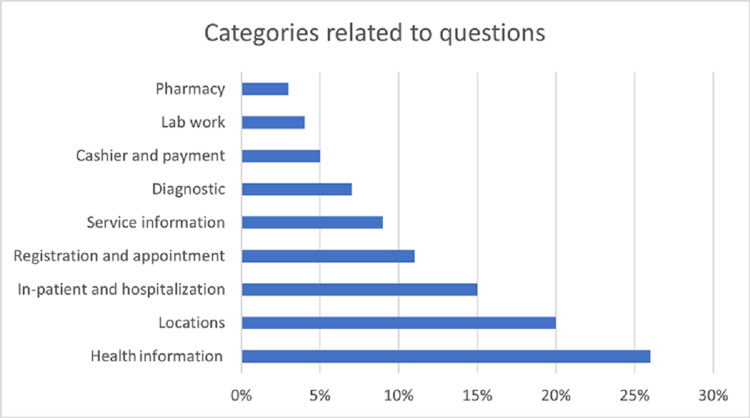
Categorical ranking of information inquiry answered successfully during the visit

Among the successful encounters, Figure 2 depicts the categorical interests that visitors displayed. Twenty-seven percent of visitors were using the robot to seek medical information. Questions such as "what is a hernia?" and "tell me something about diabetes?" were common in the question pool. Twenty percent of questions asked for directions to specialty clinic locations, such as "can you direct me to the pediatric clinic?" and "where is radiology?" Some of the requests were for information regarding hospitalization and getting appointments for outpatient visits. The questions included "do you provide registration service for surgery clinic?" and "I was admitted to the hospital; what am I supposed to do now?" Questions such as "does the hospital provide hepatitis vaccination?" were typical for the type of inquiry for services available at the hospital. Diagnostic and lab work inquiries were questions related to interpreting reports similar to "my blood sugar is 180, what does it mean?" Inquiries for insurance and co-pay were dominating in the cashier and payment category, and lastly, medication-related questions were recorded among the questions to complete the list.

In terms of service accuracy, all questions considered successfully answered were analyzed manually to ensure a meaningful response by the IGR. When comparing the intelligence data automated output by the robot and manual calibration of the performance results, there were unmatched outcomes between the two calculations. The manufacturer claimed the IGR accuracy rate in answering business-related questions was near perfect. However, manually, the calculated accuracy rate was 92.32% among the successfully answered inquiries.

## Discussion

The size of tertiary hospitals in China is humongous compared to that in the United States [5]. Any central health care institute would easily have over a thousand beds. However, outpatient clinic services remain a part of hospital operations. Due to the imbalance of medical resources, patients trust reputable hospitals for care, which leads to overwhelming outpatient traffic [6]. As a comprehensive hospital that bears the responsibility to provide care of complex medical treatment in the city, the hospital is committed to developing information technology to serve the public better.

The finding indicated that using human-computer interaction technology to provide patients with efficient hospital location consultation, hospital business process guidance, specialty clinic recommendations, and health education could reduce significant workload in outpatient clinic settings. The additional implementation of IGR helps to share the burden of human-delivered repetitive consultation work. Thus, the implementation of the IGR can help hospitals improve operational efficiency and patient experience during an outpatient visit.

The implementation of IGR was initially to improve the guidance service in the outpatient clinic. However, patients were highly interested in learning the basic medical and health knowledge that the IGR could provide. This finding was unexpected. Improving knowledge content in future IGR model designs can significantly improve the utilization at the outpatient clinic to promote and narrow the gaps of health literacy in the community.

Optimization of the outpatient guidance in the busy outpatient clinic is vital to improving the overall patient experience [7]. Therefore, information technology-driven analysis to optimize patient experience and care process are critical to achieving the intend to improve appointment registration, consultation triage, location navigation, health education, and other consultation processes of services.

Lastly, the profound impact of the coronavirus disease 2019 (COVID-19) pandemic has changed outpatient clinic practice [8]. Therefore, the design and development of robotic technology in health care should not be limited to intelligent guidance and the broadcast of preventive measures during the pandemic but, furthermore, to educate, comfort, and sometimes entertain the isolated and frighten patients.

## Conclusions

Information technology is one of the fastest-growing components in the health care industry. New analytic methodologies enable process changes that dramatically change health care delivery in various settings, both environmentally and clinically. The era of artificial intelligence will inevitably bring artificial intelligence to outpatient medicine, and the application of medical artificial intelligence technology represented by IGR is the beginning trend. The application of IGR in hospital outpatient clinics brings more convenient and efficient medical service to patients and improves service quality and patient experience. However, there is still much room for improvement in service accuracy and needs to further expand business and education functions according to the needs of patients. In addition, increased human-machine interaction enhances the patient experience and helps in the development of an innovative hospital. With the continuous improvement of intelligent referral service information systems, both humans and machines will transition from the information to the intelligent stage and improve the quality of patient care at all levels of service.

Many people continue to think that all illnesses must be treated by doctors and in health care facilities. However, some 80% of health-related problems are treatable at home. The public has little knowledge in determining when a health care provider’s help is needed or not. Therefore, enhancing the IGR education function at outpatient clinics will help to broadcast valuable health knowledge to improve health literacy in the public domain. Above all else, helping the public with health decisions can reduce the burden of outpatient clinic traffic and provide patients with information to make sound judgments about their health.
